# Genetic Characterization of Small Ruminant Lentiviruses Isolated from Dairy Sheep in Greece

**DOI:** 10.3390/v16040547

**Published:** 2024-03-31

**Authors:** Aphrodite I. Kalogianni, Ilias Bouzalas, Sofia Marka, Maria-Eleftheria Zografaki, Sofia Mavrikou, Athanasios I. Gelasakis

**Affiliations:** 1 Laboratory of Anatomy and Physiology of Farm Animals, Department of Animal Science, School of Animal Biosciences, Agricultural University of Athens (AUA), Iera Odos 75 Str., 11855 Athens, Greece; gelasakis@aua.gr; 2 Veterinary Research Institute, Hellenic Agricultural Organization-DEMETER, Campus of Thermi, 57001 Thessaloniki, Greece; bouzalas@elgo.gr; 3 Laboratory of Cell Technology, Department of Biotechnology, School of Applied Biology and Biotechnology, Agricultural University of Athens (AUA), EU-CONEXUS European University, 11855 Athens, Greece; smarka@aua.gr (S.M.); mzografaki@aua.gr (M.-E.Z.); sophie_mav@aua.gr (S.M.)

**Keywords:** small ruminant lentiviruses, sheep, Greece, genetic characterization, phylogenetic analyses, immunodominant epitopes, capsid protein

## Abstract

The high genetic heterogeneity of small ruminant lentiviruses (SRLV) renders the genetic characterization of the circulating strains crucial for the epidemiological investigation and the designation of effective diagnostic tools. In Greece, research data regarding the genetic diversity of the circulating SRLV strains is scarce, hindering the implementation of efficient surveillance and control programs. The objective of the study was to genetically characterize SRLV strains isolated from intensive dairy sheep farms in Greece and evaluate the variability of the immunodominant regions of the capsid protein. For this reason, a total of 12 SRLV-infected animals from four intensive dairy sheep farms with purebred Chios and Lacaune ewes were used for the amplification and sequencing of an 800 bp *gag*-*pol* fragment. The phylogenetic analyses revealed a breed-related circulation of strains; Chios ewes were infected with strains belonging exclusively to a separate group of genotype A, whereas strains belonging to subtype B2 were isolated from Lacaune ewes. Immunodominant epitopes of capsid protein were quite conserved among the strains of the same genotype, except for the Major Homology Region which showed some unique mutations with potential effects on viral evolution. The present study contributes to the extension of the current knowledge regarding the genetic diversity of SRLV strains circulating in sheep in Greece. However, broader genetic characterization studies are warranted for the exploration of possible recombinant events and the more comprehensive classification of the circulating strains.

## 1. Introduction

The Maedi-visna virus (MVV) and caprine arthritis-encephalitis virus (CAEV) belong to small ruminant lentiviruses (SRLV), a subgroup of viruses of the family *Retroviridae* which cause persistent and insidious infections in sheep and goats [1]. These infections can lead to the manifestation of clinical signs such as pneumonia, mastitis, arthritis, and encephalitis [2], compromising health, welfare, and productivity of the affected animals. The global spreading of SRLV infections renders their control imperative for the limitation of the monetary losses in the small ruminant industry. Considering the lack of effective treatment and vaccines against SRLV infections, the eradication of SRLV infections is based on the early diagnosis and the implementation of general management practices and biosecurity measures to prevent the spread of the virus between and within herds [3]. 

Nevertheless, the development of a “gold standard” assay for the early diagnosis of SRLV infections is a challenging endeavor considering the high genetic variability of the virus [4]. The genetic characterization of the circulating SRLV strains is an important prerequisite for the implementation of efficient surveillance and eradication programs, not only for the “mapping” of the spatial spreading of various strains, but also for the development of sensitive and specific diagnostic tools for application on a case-specific basis. For this reason, many countries have attempted the molecular characterization of the circulating SRLV strains; the phylogenetic analyses, genotyping, and classification of the isolates have identified five genotypes (A–E) and many subtypes thereof in sheep and goats [5,6,7,8,9,10]. Among them, genotypes A and B have a global spread and have been initially isolated from sheep and goats, respectively, although cross-species transmission has been previously reported in many countries [11,12,13]. On the other hand, genotypes C, D, and E have been identified in specific regions and species, and their spreading is considered more restricted compared to genotypes A and B [7,9,14,15].

Phylogenetic analyses contribute significantly to the investigation of the SRLV genetic diversity, the tracing of transmission patterns, and the assessment of the genetic distance and relationships among different viral isolates. The commonly targeted genomic regions for phylogenetic analyses in SRLV are the three major coding regions of the SRLV genome, namely, *gag*, *pol*, and *env* genes, as well as the non-coding LTR regions [8]. Among these genomic regions, the *gag* gene, which encodes structural proteins including the matrix (p16MA), capsid (p25CA), and nucleocapsid (p14NC) proteins, is highly preferred due to its conserved function. The viral capsid protein contains three immunodominant epitopes, namely, epitope 2, including the double glycine “GG” motif; epitope 3; and the Major Homology Region (MHR), which determine the genetic variability between the SRLV strains [16]. Considering that the viral capsid protein is commonly used as an antigen for the detection of SRLV-specific antibodies [17,18,19], the high divergence in linear epitopes may adversely affect the diagnostic performance of serological diagnostic tools [7]. 

Although SRLV infections have been previously reported in Greece [20,21,22,23], relevant research data regarding circulating strains and their genetic heterogeneity are scarce [24,25,26]. Hence, the objective of this study was the molecular characterization of SRLV strains isolated from dairy sheep in Greece based on the partial characterization of the *gag*-*pol* genomic region, with the genetic analyses presented including phylogenetic analyses and heterogeneity of linear epitopes of capsid protein between the different strains. 

## 2. Materials and Methods

### 2.1. Animals, Blood Samples, and DNA Extraction

A total of seven ewes that were constantly found seropositive and 13 ewes that were constantly found seronegative with a commercial indirect ELISA test (ELISA, CAEV/MVV Total Ab Test, IDEXX, Westbrook, ME, USA) during a two-year epidemiological study conducted in four intensive dairy sheep farms in Greece [23,27] were used for the purpose of the present study. From each animal, a whole blood sample (~9 mL) was collected from the jugular vein in an EDTA-anticoagulated tube and was further processed for the molecular analysis. The protocol of this study was approved by the Animal Research Ethics Committee of the Agricultural University of Athens following the national animal welfare regulations (protocol no 9).

Leukocyte pellets were isolated from whole blood samples. A total of 2 mL blood was mixed with 13 mL of ACK lysis buffer (8.02 g NH_4_Cl, 0.84 g NaHCO_3_, and 0.37 g EDTA per liter, pH 7.2–7.4) in a Falcon^TM^ 15 mL conical centrifuge tube. After 15 min of incubation at room temperature, the mixtures were centrifuged at 450× *g* for 15 min. The supernatant was discarded, and the leukocyte pellets were diluted in 1 mL ACK lysis buffer and transferred in 2 mL microcentrifuge tubes. The mixtures were centrifuged at 450× *g* for 4 min and the supernatant was discarded. The leukocyte pellets were resuspended in 1 mL PBS (phosphate buffered saline, pH = 7.4), and were centrifuged at 650× *g* for 4 min. The leukocyte pellets were used for the genomic DNA extraction with a commercial kit (PureLink^®^ Genomic DNA Kit, Life technologies Corp., Carlsbad, CA, USA), according to the manufacturer’s instructions. The procedure of DNA extraction was based on the selective binding of DNA to a silica-based membrane in the presence of chaotropic salts (ethanol and guanidinium hydrochloride). DNA concentration and purity were measured in a spectrophotometer (Q5000, Quawell Technology, Inc., San Jose, CA, USA) considering A260/A280 and A260/A230 ratios. Samples with A260/280 and A260/230 ratios of ~1.8 and 2.0–2.2, respectively, were considered as pure for DNA and acceptable for PCR and sequencing. DNA samples were stored at −20 °C until they were ready to be assayed. 

### 2.2. PCR, Sequencing, and Phylogenetic Analyses

A nested PCR protocol, previously described by Grego et al., 2007 [7], was used after a slight modification. In brief, the first round of PCR amplified a 1.3 kb DNA fragment of the *gag*-*pol* region. The reaction was carried out in 50 μL final volume which contained a 5 μL DNA sample (~1 μg), 400 nM of each primer (F1: TGGTGARKCTAGMTAGAGACATG; R1: CATAGGRGGHGCGGACGGCASCA), 25 μL of OneTaq 2× Master Mix (New England, Biolabs Inc., Hitchin, UK), and 18 μL of DNase-free water. The amplification protocol consisted of initial denaturation at 94 °C for 5 min, followed by 40 cycles of denaturation at 95 °C for 30 s, annealing at 55 °C for 30 s, and extension at 72 °C for 1.5 min, terminating with a final extension at 72 °C for 5 min. The second round of PCR was carried out with 4 μL from PCR amplicons from the first PCR and a different primer set (F2: CAAACWGTRGCAATGCAGCATGG; R2: GCGGACGGCASCACACG) for the amplification of a ~800 bp *gag*-*pol* gene fragment (988–1802 positions in genome of reference strain K1514 with GenBank accession number M60609). The amplification protocol of the second PCR consisted of initial denaturation at 94 °C for 5 min, followed by 40 cycles of denaturation at 95 °C for 20 s, annealing at 60 °C for 30 s, and extension at 72 °C for 1.5 min, terminating with a final extension at 72 °C for 5 min. All amplifications were carried out in the same thermal cycler (Labcycler, Sensoquest GmbH, Göttingen, Germany) in duplicate, and a negative control reaction with DNase-free water instead of DNA template was used each time to determine any possible nucleic acid contamination. All PCR products were mixed with DNA Gel Loading Dye (Invitrogen, Vilnius, Lithuania) and were electrophoresed in 1.5% agarose gel in 1× RNase-free TAE buffer (Invitrogen, Vilnius, Lithuania), stained with SYBRsafe DNA gel staining (Invitrogen, Carlsbad, CA, USA), and visualized under ultraviolet (UV) light. For the evaluation of the amplicon length, a 100 bp ladder was used (GeneRuler 100 bp DNA Ladder, Thermoscientific, Vilnius, Lithuania).

PCR bands of desired length were gel-extracted (PureLink™ Quick Gel Extraction Kit, Invitrogen, Vilnius, Lithuania) and sequenced in both directions with Sanger dideoxy sequencing method on an ABI PRISM 3730xl Genetic Analyzer in an external laboratory (Cemia SA, Larissa, Greece) using the primers F2 and R2. 

All the phylogenetic analyses were performed using the MEGA11 software [28]. The nucleotide sequences were trimmed according to the obtained chromatographs and the consensus sequences generated were submitted to GenBank with accession numbers OR283217–OR283228. After this, a similarity search was conducted with the NCBI (National Center of Biotechnology Information) database using BLAST (Basic Local Alignment Search Tool), and the sequences most homologous to them and all the representative sequences from SRLV strains of genotypes A, B, C, and E from different geographical regions were aligned by CLUSTAL W [29]. After the alignment, pairwise genetic distances were calculated with the p-distance model applying the gamma distribution parameter between the nucleotide sequences. A phylogenetic tree was constructed using the Maximum Likelihood (ML) method [30] and the General Time Reversiblemodel with gamma distribution (G) and invariant sites (I) with 100 bootstrap replicates [31] based on the lowest Bayesian Information Criterion (BIC) [32]. 

Also, nucleotide sequences were translated into amino acid sequences and aligned using the MEGA v.11 software, for the calculation of their pairwise genetic distance (p-distance with gamma distribution parameter). Also, the immunodominant regions of gag protein (epitope 2, epitope 3, and MHR) were comparatively assessed.

## 3. Results

### 3.1. Pairwise Nucletide and Amino Acid Sequence Comparisons

A total of 12 animals were found positive with the nested PCR protocol, and their PCR products were sequenced. In Table 1, information about the breed, age, and serological status of these animals, the accession number of the respective sequences, the farms, and their geographical location are summarized. All PCR negative animals (8/20) were constantly seronegative during the study.

The mean pairwise genetic distance of partial nucleotide *gag*-*pol* sequences obtained in this study was 15.0% and varied from 1.2% between the s-8m and s-98m sequences to 29.2% between the s-40sx and s-42fl sequences (Table 2). Also, the intra-farm pairwise comparisons were 19.0% for farm A (s-16sx, s-56sx, and s-40sx sequences), 5.0% for farm B (s-11s, s-14s, s-35s, and s-83s sequences), 7.0% for farm C (s-10fl, s-21fl, and s-42fl sequences), and 1.2% for farm D (s-8m and s-98m, sequences), with an average distance of 8.1% (inter-farm mean genetic distance).

The overall mean pairwise genetic distance of partial *gag*-*pol* sequences from the present study, the SRLV representative reference strains, and the strains isolated from nearby countries (Italy, Turkey, Jordan, and Lebanon) was 24.0%. As presented in Table 3, *gag*-*pol* sequences from the present study presented the highest genetic distance with the 1GA strain (genotype C) (23.5–32.2%), and the Roccaverano and Seui strains (genotype E) (35.5–41.4%). The strains s-56sx, s-11s, s-14s, s-35s, s-83s, s-10fl, s-21fl, and s-42fl were the most similar to the Greek strains SRLV-Greece-S1 and SRLV-Greece-S2 (genotype A) (genetic distance 9.1–12.6%), whereas the strains s-16sx, s-40sx, s-8m, and s-98m were most related to Italian strains SRLV001, SRLV042, lt-Pi1, and lt-007 of genotype B2 (genetic distance 6.2–8.9%).

### 3.2. Phylogenetic Analysis

**Figure 1:**
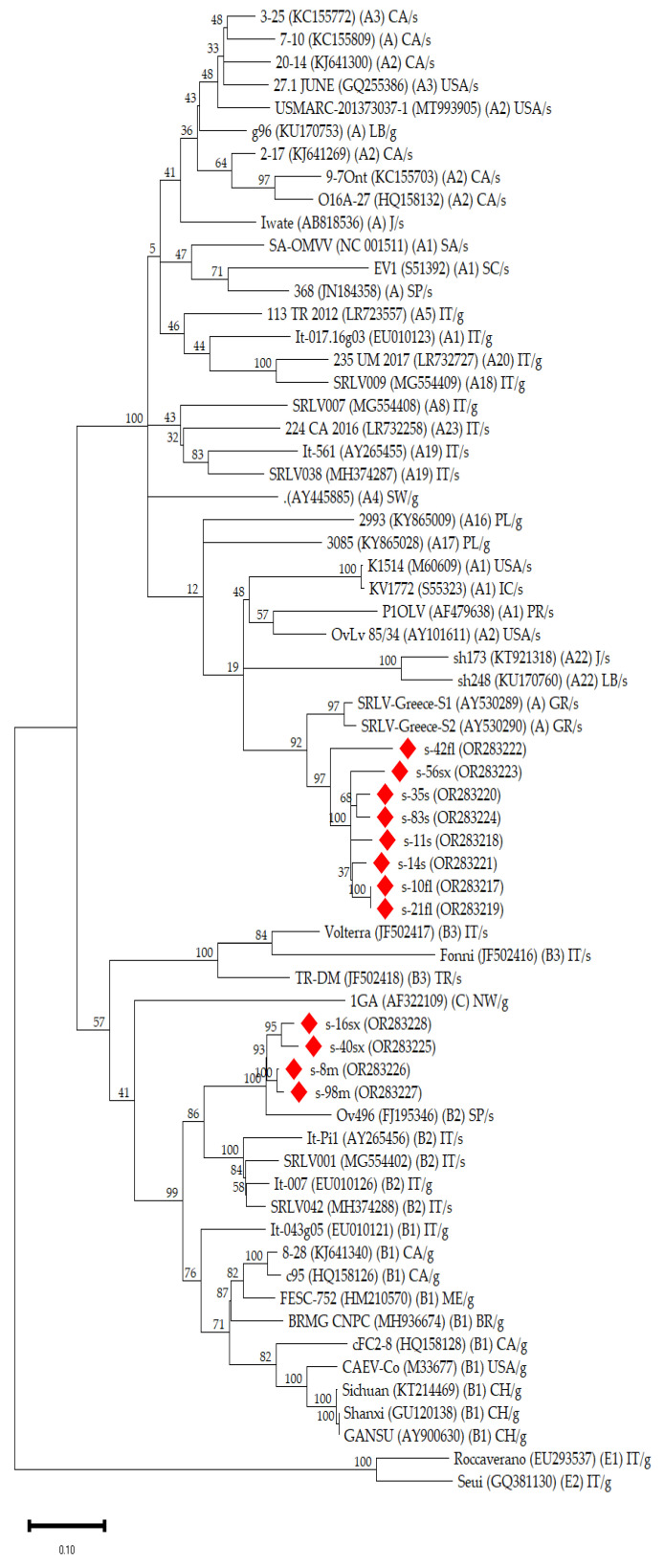
Phylogenetic tree indicates the relationship of *gag* nucleotide sequences (~800 bp) of the present study with the available database (Genbank) SRLV strains originating from different geographical areas. This unrooted tree was inferred in MEGA11 by using the Maximum Likelihood method and General Time Reversible. Bootstrap values are based on 100 repetitions and are shown at the nodes. The tree with the highest log likelihood (−16884.54) is shown. The percentage of trees in which the associated taxa clustered together is shown above the branches. A discrete Gamma distribution was used to model evolutionary rate differences among sites [5 categories (+*G*, parameter = 0.6938)]. The rate variation model allowed for some sites to be evolutionarily invariable ([+*I*], 17.37% sites). This analysis involved 65 nucleotide sequences. Sequences from the present study are shown with a red rhombus symbol (GenBank: OR283217 to OR283228). Database derived sequences are denoted with their strain name, GenBank accession number and genotype in parenthesis, the abbreviation of the country (CH: China; BR: Brazil; CA: Canada; GR: Greece; IC: Iceland; IT: Italy; J: Jordan; LB: Lebanon; ME: Mexico; NW: Norway; PL: Poland; PR: Portugal; SA: South-Africa; SC: Scotland; SP: Spain; SW; Switzerland; TR: Turkey; USA: United States of America), and the host (s: sheep; g: goat).

The *gag*-*pol* sequences isolated in the present study were clustered in two groups in the phylogenetic tree (Figure 1). The first group included s-8m, s-98m, s-16sx, and s-40sx sequences and was strongly associated with the Spanish (FJ195346) and the Italian (MG554402, EU010126, AY265456, and MH374288) strains of genotype B2. The other group included s-10fl, s-21fl, s-42fl, s-11s, s-14s, s-35s, s-83s, and s-56sx sequences and was clustered with other Greek (AY530289 and AY530290) strains of genotype A. 

### 3.3. Comparative Assessment of immunodominant Linear Epitopes of Capsid Protein

**Figure 2:**
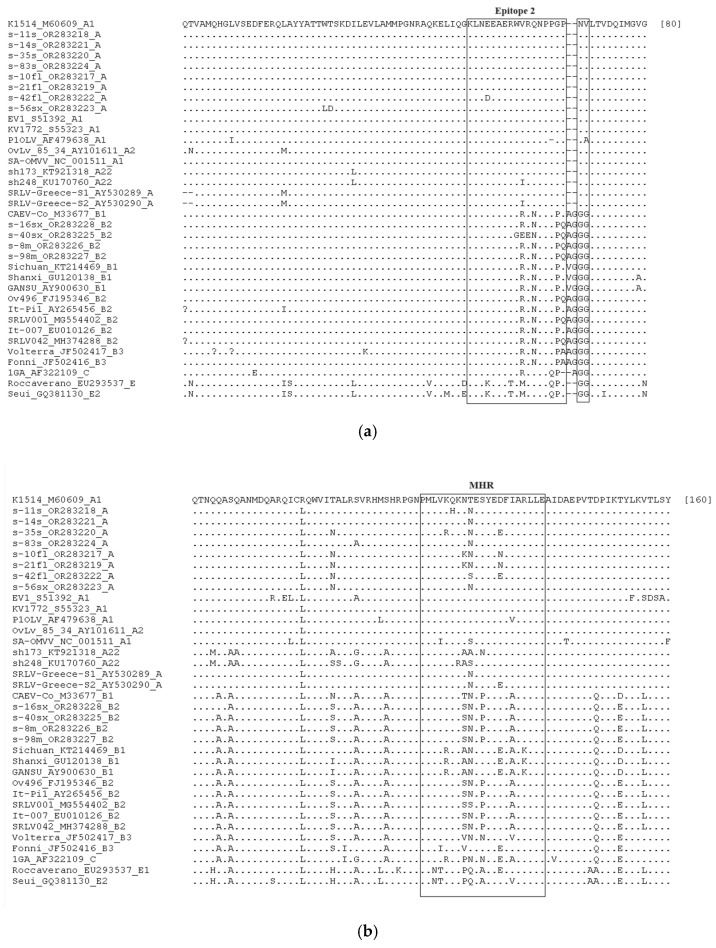

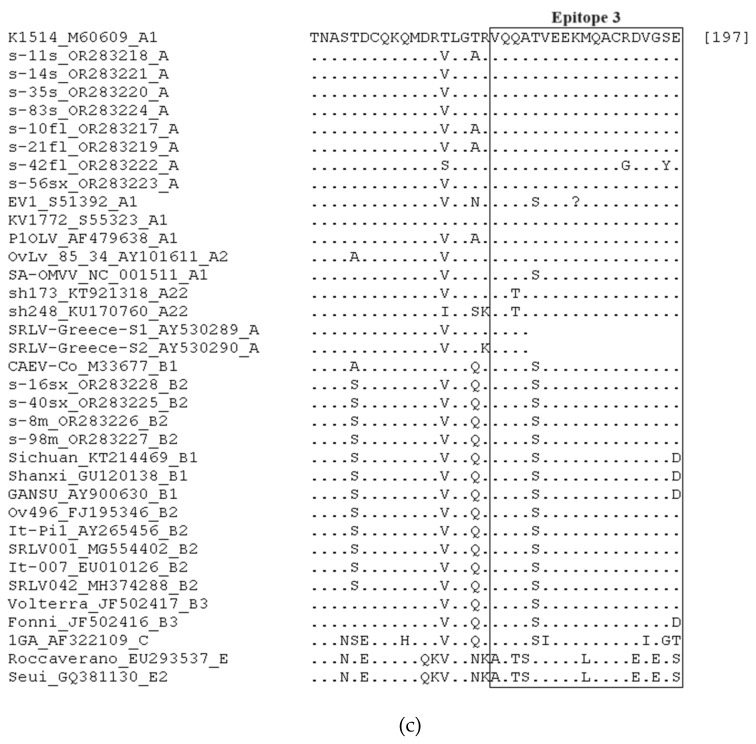
Amino acid sequence alignment of *gag*-*pol* sequences from the present study (OR283217–OR283228) and reference SRLV strains from GenBank belonging to genotypes A, B, C, and E. Immunodominant regions are boxed: epitope 2 and “GG” motif (**a**), Major Homology Region (**b**), and epitope 3 (**c**). Dots represent identical amino acid residues with reference strain K1514, dashes indicate deletions, and missing information sites are denoted by a question mark.

In Figure 2a–c, the alignment of the amino acid sequences is presented, and epitope 2, including the double glycine “GG” motif; epitope 3; and the MHR are comparatively assessed. Strains from the present study belonging to genotype A, according to the afore-mentioned phylogenetic tree, demonstrated high conservation regarding epitopes 2 and 3, whereas more alterations were found in MHR. Also, all strains belonging to genotype A presented the asparagine-valine “NV” motif, whereas the strains of genotype B presented the typical double glycine “GG” motif. Also, in the s-42fl strain, glutamic acid (E) was replaced by aspartic acid (D) in epitope 2, and arginine (R) and serine (S) were replaced by glycine (G) and tyrosine (Y), respectively, in epitope 3. In the MHR, i) asparagine (N) was replaced by serine (S) in the s-42fl strain and by lysine (K) in the s-10fl and s-21fl strains; ii) aspartic acid (D) was replaced by asparagine (N) in the s-10fl and s-21fl strains, and by glutamic acid (E) in the s-35s and s-42fl strains; iii) glutamine (Q) was replaced by histidine (H) in s-11s strains; and iv) lysine (K) was replaced by arginine (R) in s-35s strains. Strains from the present study belonging to genotype B2 presented identical amino acid residues in all epitopes, except for the s-40sx strain in epitope 2, where 3 amino acids were substituted: tryptophan (W) with glycine (G), and the next two consecutive arginines (R) with two glutamic acids (E). Also, strains from the present study belonging to genotype A demonstrated high similarity with a few discrepancies with the reference strains of the same genotype and with the strains that presented low genetic distance in the phylogenetic tree, while strains from the present study belonging to genotype B2 demonstrated absolute identity with the other reference strains belonging to the same genotype, except for the s-40sx strain. 

## 4. Discussion

This is the first time that an extended *gag*-*pol* fragment (~800 bp), including all the immunodominant regions of capsid protein, from SRLV strains isolated in Greece is analyzed for their genetic characterization. Although numerous sequencing and phylogenetic analyses have been performed for the genotyping and classification of SRLV strains worldwide, based on different regions of the viral genome (mainly *gag*, *pol*, and *env* genes) [5,6,7,8,9,13,33,34,35,36,37,38,39], phylogenetic data regarding circulating SRLV strains in Greece are limited and derived from previous studies based on partial *pol* (~390 bp) [25,26] and *gag* (574 bp) [24] sequences, which in the latter case did not include all the immunodominant regions of capsid protein in contrast to our study.

In the present study, all the seropositive animals also tested PCR positive (infected seropositive animals), while five seronegative animals were found PCR positive (infected seronegative animals), indicating the significance of the combination of serological and molecular testing for the detection of SRLV-infected animals [25,40,41,42,43]. Infected seronegative animals were found in both breeds and on all farms. This underpins the possibility of late seroconversion rather than poor diagnostic performance of the serological test due to the circulation strains with antigenic variation. Indeed, the studied antigenic epitopes did not present any alteration that could be linked with the putative misdiagnosed animals. This is also supported by the age distribution of the infected seropositive animals compared to the infected seronegative animals (2.0 to 7.0 vs. 1.5 to 3.0 years, respectively), which indicates the underdiagnosis of the younger animals by using ELISA.

The phylogenetic analysis in the present study was based on the *gag*-*pol* region of the viral genome, which is considered relatively conserved (more conserved than the *env* and less conserved than the *pol* regions) and suitable for designation of PCR primers, sequencing, and meaningful comparisons between different sequences [18,44]. At the same time, this region encodes for significant viral structural (*gag* gene) and functional (*pol* gene) proteins and is under selective pressure presenting stable genetic divergence for phylogenetic analyses and genetic characterization of the isolates. Also, in the present study the size of *gag*-*pol* gene fragment was ~800 bp, making it suitable for both sequencing and phylogenetic analysis, as it provides enough sequence information while being manageable in terms of sequencing efforts and computational analysis. The phylogenetic analyses in the present study revealed the circulation of strains belonging to genotypes A and B (subtype B2) in the studied farms. This is consistent with previous studies that have also reported the existence of strains belonging to genotypes A [24,25,45] and B [25] in Greece. Specifically, phylogenetic analyses of partial *gag* and LTR sequences identified strains more similar to MMV-like strains (A genotype) [24,45], whereas strains from both genotypes A and B were identified with phylogenetic analyses based on partial *pol* sequences [25].

*Gag*-*pol* sequences from our study were clustered into two discrete groups. The sequences from the first group were grouped within the same clade as the strains previously isolated in Greece [24], although the latter were shorter sequences including a 574-nucleotide fragment of the *gag* gene. Also, the sequences from the present study were closely related to the strains isolated from the nearby countries of the Middle East (Lebanon and Jordan) (subtypes A22–A24) (see Figure 1). The genetic distance between the sequences from the present study and the other Greek strains was lower than 15.0%, whereas it was greater than 15.0% in the case of sequences from subtypes A22–A24. Considering that a 15% genetic distance is considered the limit for the categorization of variants in the same subtype [8], it could be suggested that this indicates the identification of a new subtype into the genotype A, including the Greek strains. On the other hand, the other group of *gag*-*pol* sequences was clearly clustered with B2 strains isolated in Italy and Spain [7,46,47,48], presenting very low values of nucleotide distance (<10%) as derived from the nucleotide pairwise comparisons. The genetic association of SRLV strains of genotypes A and B with these specific regions is consistent with previous phylogenetic studies indicating the Middle East and Central Europe as the ancestral regions of the A and B genotypes, respectively [49,50]. Nevertheless, further investigation and bioinformatics analyses of whole-genome sequence data are imperative for the exploration of the origin of SRLV strains isolated in the present study.

Our results also confirmed the infection of sheep with strains of genotype B, which were initially isolated from goats [50]. This finding is in agreement with previous studies, which reported cross-species transmission of A and B genotype strains between sheep and goats [11,12,13]. In particular, subtype B2, which was found in our study, has been reported in sheep in several studies in the past [7,8,46,47]. Moreover, the results obtained from the constructed phylogenetic tree indicated the circulation of strains of both A and B2 genotypes only in the single mixed farm (farm A), where both Chios and Lacaune sheep were reared. On the other hand, in Chios farms (farms B and C), all the sequences were associated with strains of genotype A, whereas the Lacaune farm (farm D) sequences were associated with strains of subtype B2. This is in accordance with the results from mean pairwise genetic distances between the sequences; all sequences originating from the same farm presented low genetic heterogeneity < 15.0%, except for the *gag*-*pol* sequences in farm A (19.0%). Also, the identification of strains of genotypes A and B2 in Chios and Lacaune sheep, respectively, suggests a breed-related circulation of strains that may be associated with the origin of animals. Considering that the Chios breed is an indigenous Greek breed with a limited number of breeding stocks across the country, and Lacaune breeding stocks have been massively imported during the last decades from France, the circulation of strains with high genetic similarity among the sheep of the same breed is possibly related to their common origin. This is also confirmed by the fact that in farm A, where Lacaune and Chios sheep were housed together, genotype A was identified only in Chios sheep and genotype B2 only in Lacaune sheep, implying that the initial breeding animals were infected when introduced into the farm, and then their lambs were vertically infected with the same viral strain. However, the possibility of breed-related infectivity among the different SRLV genotypes cannot be excluded. These assumptions need further investigation which must include the molecular characterization of strains isolated from a large number of animals from this farm to explore the possibility of a recombinant event characterizing more viral genomic regions.

Immunodominant epitopes of capsid protein have been previously characterized in many studies for the assessment of their heterogeneity [6,7,9,24,34,51,52,53,54]. Considering that immunodominant linear epitopes of capsid protein are strongly associated with the type-specific host immune response [46,53,55], their role in serological diagnosis is crucial. The investigation of genetic heterogeneity in these epitopes among the different SRLV strains could reveal diagnostic drawbacks of the existent serological diagnostic tools and contribute to the development of highly efficient ones [56,57]. Also, serotyping diagnostic tools discriminating the different SRLV genotypes have been developed using subunits of the viral capsid protein [58,59]. These tests could be used for the implementation of epidemiological studies in countries where the genetic characterization of the circulating SRLV strains has not been performed. Based on this, the exploration of immunodominant linear epitopes of capsid protein in SRLV strains in our country could be exploited for the development of highly sensitive serological tests and serotyping tools instead of the costly and time-consuming genetic analyses. Additionally, it is the first time that a comparative analysis of all the immunodominant linear epitopes of capsid protein is performed in SRLV sequences isolated in Greece. A previous study in our country analyzed shorter amino acid sequences, including only epitope 2 and the MHR [24], without, however, comparing the specific regions of epitopes, instead comparing the total amino acid sequences. Our findings confirmed the presence of the asparagine-valine “NV” and the double glycine “GG” motifs clustered in genotypes A and B, respectively, as previously described in similar studies [34,39,49,54,60,61]. Also, epitopes 2 and 3 were found highly conserved when the strains of genotype A from the present study were compared to the reference strains of the same clade, with the exception of one isolate (s-42fl) in which three new amino acid replacements were identified [one conservative substitution in epitope 2 (D/E) and two non-conservative substitutions in epitope 3 (G/R and Y/S)]. Regarding MHR, in our study, medium heterogeneity was presented among the sequences belonging to genotype A, whereas three new replacements were also observed (H/Q, N/D, and K/N). This finding is in accordance with previous studies, which reported moderate variability in the MHR of isolated strains [39,51,60]. Even though genotype A strains in our study demonstrated high similarity with the strains previously isolated in Greece, the new unique replacements that were identified could lead to a significant divergence related either to a poor diagnostic performance of serological diagnostic tools based on specific epitopes or to alternative infectivity. On the other hand, strains belonging to subtype B2 demonstrated absolute similarity in all epitopes with the reference strains of this subtype, apart from one isolate that demonstrated a specific triple amino acid substitution in epitope 2 (GEE/WRR).

Although it is the first time that the genetic characterization of SRLV isolates from our county included both phylogenetic analyses and assessment of heterogeneity of linear epitopes of capsid protein, the number of the studied animals does not allow the generalization of the results. However, our findings facilitate the extraction of useful conclusions regarding the epidemiology of SRLV infections, as the farms used in the study (i) are representative of the intensive farming system, (ii) are located in different counties of Greece with well-developed dairy sheep farming sectors, (iii) are rearing purebred sheep of the most commonly used dairy sheep breeds in the country (one indigenous and one imported breed). Genetic characterization of different and more extended viral genomic regions or even whole-genome sequencing of isolated strains are warranted for the confirmation of the results from the present study and the comprehensive classification of strains. Also, further studies including both sheep and goats of various breeds and regions of Greece could contribute to (i) the extension of the current knowledge regarding the genetic variability of SRLV strains circulating in different breeds reared under various farming systems in the country, (ii) the elucidation of viral evolution, (iii) the exploration of possible recombination events, and (iv) the designation of effective surveillance and eradication programs against SRLV infections.

## 5. Conclusions

The present study constitutes the first partial genetic characterization of SRLV strains circulating in dairy sheep in Greece based on an extended *gag*-*pol* fragment including all the immunodominant regions of the capsid protein. The phylogenetic analysis revealed the circulation of strains belonging to genotype A, clustered in a distinct clade with the other Greek isolates, and strains grouped clearly with strains of subtype B2. Chios sheep were found to be infected with A genotype, whereas Lacaune sheep were infected with B2 strains, implying a breed-associated circulation of strains and a specific transmission pattern possibly related to the available breeding stocks. Even though amino acid sequences of immunodominant epitopes of capsid protein were sufficiently conserved, especially in B2 strains, remarkable mutations were found in the MHR of A strains, potentially affecting their virulence or challenging serological diagnosis.

## Figures and Tables

**Table 1:** Information of the PCR positive samples of the study.

Sample ID	Breed	Age (Years)	Serological Status	Farm	Location	Accession Number	Genotype
s-16sx	Lacaune	3.5	+	A	Central Greece	OR283228	B
s-40sx	Lacaune	2.0	−	A		OR283225	B
s-56sx	Chios	7.0	+	A		OR283223	A
s-11s	Chios	2.5	+	B	Epirus	OR283218	A
s-14s	Chios	7.0	+	B		OR283221	A
s-35s	Chios	1.5	−	B		OR283220	A
s-83s	Chios	7.0	+	B		OR283224	A
s-10fl	Chios	4.0	+	C	Peloponnese	OR283217	A
s-21fl	Chios	3.0	−	C		OR283219	A
s-42fl	Chios	3.0	−	C		OR283222	A
s-8m	Lacaune	3.0	−	D	Western Greece	OR283226	B
s-98m	Lacaune	3.0	+	D		OR283227	B

+: ELISA positive animal; −: ELISA negative animal.

**Table 2:** Nucleotide (plain numbers) and amino acid (bold numbers) distance values (%) between partial *gag*-*pol* sequences obtained in this study.

	s-16sx	s-40sx	s-56sx	s-11s	s-14s	s-35s	s-83s	s-10fl	s-21fl	s-42fl	s-8m
**s-40sx**	3.9	-	-	-	-	-	-	-	-	-	-
	**2.2**	**-**	**-**	**-**	**-**	**-**	**-**	**-**	**-**	**-**	**-**
**s-56sx**	26.8	27.3	-	-	-	-	-	-	-	-	-
	**13.9**	**15.2**	**-**	**-**	**-**	**-**	**-**	**-**	**-**	**-**	**-**
**s-11s**	24.2	24.9	6.8	-	-	-	-	-	-	-	-
	**14.6**	**15.8**	**3.8**	**-**	**-**	**-**	**-**	**-**	**-**	**-**	**-**
**s-14s**	24.7	25.2	5.4	4.7	-	-	-	-	-	-	-
	**13.0**	**14.2**	**2.5**	**2.0**	**-**	**-**	**-**	**-**	**-**	**-**	**-**
**s-35s**	24.7	25.0	5.7	5.2	4.8	-	-	-	-	-	-
	**13.8**	**15.0**	**3.4**	**3.2**	**2.0**	**-**	-	-	-	-	-
**s-83s**	23.0	24.3	4.7	4.7	4.7	3.6	-	-	-	-	-
	**12.6**	**13.8**	**3.4**	**3.2**	**2.0**	**2.4**	**-**	**-**	**-**	**-**	**-**
**s-10fl**	25.4	26.1	6.5	5.1	4.1	4.7	5.1	-	-	-	-
	**14.2**	**15.4**	**3.8**	**2.8**	**2.4**	**2.4**	**3.6**	**-**	**-**	**-**	**-**
**s-21fl**	25.4	26.1	6.5	5.1	4.1	4.7	5.1	-	-	-	-
	**14.2**	**15.4**	**3.8**	**2.8**	**2.4**	**2.4**	**3.6**	**-**	**-**	**-**	**-**
**s-42fl**	28.9	29.2	11.9	11.6	10.5	11.2	11.4	11.1	11.1	**-**	**-**
	**15.9**	**17.1**	**5.5**	**6.1**	**4.9**	**5.3**	**6.5**	**6.1**	**6.1**	-	-
**s-8m**	4.9	5.6	25.2	23.0	24.4	23.9	22.6	24.3	24.3	26.5	-
	**2.4**	**2.8**	**14.3**	**14.6**	**13.8**	**14.6**	**13.4**	**15.0**	**15.0**	**15.9**	**-**
**s-98m**	5.6	6.3	25.6	23.2	24.4	23.8	22.3	24.4	24.4	26.4	1.2
	**2.4**	**2.8**	**14.3**	**14.3**	**13.5**	**14.3**	**13.5**	**14.7**	**14.7**	**15.9**	**0.0**

**Table 3:** Nucleotide (plain numbers) and amino acid (bold numbers) distance values (%) between partial *gag*-*pol* sequences obtained in this study and representative SRLV strains.

	s-16sx	s-40sx	s-56sx	s-11s	s-14s	s-35s	s-83s	s-10fl	s-21fl	s-42fl	s-8m	s-98m
**EV1 (A1/SC)**	27.9	29.3	24.2	21.2	22.4	21.7	21.9	22.8	22.8	24.7	26.5	25.8
	**18.7**	**19.9**	**12.2**	**13.3**	**12.9**	**13.3**	**11.2**	**14.1**	**14.1**	**15.5**	**19.1**	**18.4**
**K1514 (A1/USA)**	23.3	24.8	22.0	19.3	21.2	19.6	19.8	20.5	20.5	23.8	24.2	23.8
	**14.2**	**15.4**	**8.0**	**8.4**	**6.4**	**7.6**	**6.8**	**8.8**	**8.8**	**8.5**	**14.6**	**14.7**
**SA-OMVV (A1/SA)**	24.1	25.7	21.3	19.6	19.8	19.1	20.6	20.0	20.0	21.5	23.9	23.9
	**18.3**	**19.5**	**8.4**	**11.6**	**10.0**	**11.6**	**10.4**	**12.0**	**12.0**	**12.2**	**18.7**	**18.0**
**KV1772 (A1/IC)**	23.4	25.0	22.2	19.5	21.3	19.8	20.0	20.7	20.7	23.6	24.3	24.0
	**13.8**	**15.0**	**7.6**	**8.0**	**6.0**	**7.2**	**6.4**	**8.4**	**8.4**	**8.1**	**14.2**	**14.3**
**OvLv 85/34 (A2/USA)**	20.5	22.7	19.0	18.7	18.5	17.7	17.2	18.2	18.2	20.1	20.9	21.4
	**15.4**	**16.6**	**7.6**	**8.4**	**6.4**	**7.6**	**6.8**	**8.8**	**8.8**	**9.3**	**15.0**	**14.7**
**SRLV007 (A8/IT)**	27.3	28.9	20.7	18.6	19.8	18.8	18.1	19.6	19.6	21.0	26.2	26.2
	**17.4**	**18.6**	**10.5**	**12.8**	**11.6**	**12.8**	**12.0**	**12.0**	**12.0**	**13.8**	**18.2**	**17.6**
**SRLV009 (A18/IT)**	26.8	29.6	22.7	20.4	21.1	20.4	20.9	21.3	21.3	23.7	26.3	24.8
	**19.0**	**20.2**	**11.8**	**14.4**	**13.2**	**14.0**	**13.6**	**14.8**	**14.8**	**15.9**	**19.4**	**18.8**
**SRLV038 (A19/IT)**	25.0	26.9	20.1	18.4	18.7	17.7	19.1	17.6	17.6	19.5	24.5	24.4
	**17.4**	**18.6**	**7.1**	**10.0**	**9.2**	**10.0**	**9.6**	**10.0**	**10.0**	**11.0**	**17.8**	**17.1**
**It-561 (A19/IT)**	27.4	27.5	20.7	19.8	20.2	20.8	20.6	19.5	19.5	20.3	24.9	25.3
	**18.2**	**19.4**	**9.7**	**11.5**	**10.7**	**11.5**	**11.1**	**11.5**	**11.5**	**11.5**	**19.0**	**19.0**
**sh248 (A22/LB)**	28.3	28.7	24.2	23.5	21.9	21.5	22.4	21.7	21.7	24.0	27.4	28.5
	**15.8**	**17.0**	**11.3**	**11.6**	**10.0**	**11.6**	**10.0**	**11.2**	**11.2**	**12.6**	**17.0**	**17.1**
**sh173 (A22/J)**	30.0	30.4	26.2	24.8	23.4	22.8	24.9	23.7	23.7	25.1	28.6	28.1
	**15.8**	**17.0**	**10.5**	**10.8**	**9.6**	**11.2**	**11.2**	**11.2**	**11.2**	**11.0**	**16.6**	**16.3**
**SRLV-Greece-S1 (A/GR)**	22.4	24.2	12.6	10.0	11.5	10.0	9.6	10.4	10.4	11.1	23.1	22.1
	**10.6**	**12.2**	**2.2**	**1.7**	**0.6**	**2.2**	**1.1**	**2.8**	**2.8**	**3.3**	**11.1**	**11.1**
**SRLV-Greece-S2 (A/GR)**	22.9	25.5	12.6	10.0	11.0	10.4	9.1	10.4	10.4	11.3	23.4	22.4
	**11.7**	**13.3**	**3.9**	**3.3**	**2.2**	**2.8**	**2.8**	**3.9**	**3.9**	**3.9**	**12.2**	**12.2**
**CAEV-Co (B1/USA)**	15.1	15.0	27.2	25.2	23.9	26.1	24.8	24.9	24.9	26.0	13.2	13.1
	**8.4**	**9.2**	**14.3**	**15.3**	**13.7**	**14.1**	**14.1**	**14.5**	**14.5**	**15.5**	**7.2**	**6.5**
**GANSU (B1/CH)**	15.1	15.4	26.9	24.1	23.7	24.4	24.2	23.9	23.9	27.9	14.1	14.5
	**10.0**	**10.8**	**17.3**	**16.9**	**15.3**	**14.5**	**15.7**	**16.1**	**16.1**	**17.6**	**9.6**	**9.3**
**Ov496 (B2/SP)**	9.9	9.6	26.8	24.2	24.6	24.9	24.2	25.2	25.2	28.5	8.7	9.5
	**2.8**	**3.2**	**15.2**	**16.1**	**14.5**	**15.3**	**14.1**	**15.7**	**15.7**	**16.7**	**2.0**	**2.0**
**SRLV042 (B2/IT)**	7.4	8.5	28.1	24.9	25.7	25.4	24.5	25.9	25.9	28.3	6.2	6.9
	**10.1**	**10.5**	**17.4**	**19.4**	**19.0**	**20.2**	**18.5**	**19.8**	**19.8**	**21.7**	**9.3**	**8.5**
**It-007 (B2/IT)**	7.7	8.6	28.1	25.2	25.1	25.4	24.6	26.4	26.4	29.4	6.4	7.2
	**10.4**	**10.8**	**16.9**	**19.3**	**18.5**	**19.7**	**18.1**	**19.3**	**19.3**	**21.2**	**9.6**	**8.9**
**It-Pi1 (B2/IT)**	8.3	8.9	27.5	26.0	25.8	26.1	25.2	26.1	26.1	29.3	6.3	7.1
	**15.7**	**16.1**	**20.3**	**22.9**	**22.1**	**23.3**	**21.7**	**22.9**	**22.9**	**24.9**	**14.9**	**14.2**
**SRLV001 (B2/IT)**	8.8	8.6	27.6	25.0	26.5	26.6	25.3	27.3	27.3	29.2	6.9	7.2
	**10.8**	**11.2**	**17.7**	**19.7**	**19.3**	**20.5**	**18.9**	**20.1**	**20.1**	**21.2**	**10.0**	**9.3**
**Volterra (B3/IT)**	23.9	26.2	26.4	26.5	27.7	26.3	26.7	26.2	26.2	28.8	22.8	23.3
	**14.6**	**15.4**	**17.9**	**20.3**	**19.5**	**20.7**	**19.1**	**20.3**	**20.3**	**21.5**	**14.6**	**13.9**
**TR-DM (B3/TR)**	25.2	25.7	27.9	28.6	27.4	26.9	28.5	28.5	28.5	31.6	23.9	23.9
	**14.1**	**14.9**	**16.5**	**18.5**	**17.7**	**18.9**	**18.1**	**18.5**	**18.5**	**20.4**	**13.7**	**13.0**
**1GA (C/NW)**	25.9	25.7	32.0	32.2	30.4	31.3	30.4	31.5	31.5	31.6	23.5	24.2
	**20.6**	**21.9**	**23.3**	**25.0**	**23.8**	**23.8**	**23.8**	**24.2**	**24.2**	**25.4**	**20.6**	**20.0**
**Roccaverano (E1/IT)**	35.5	37.8	41.4	39.8	39.9	38.7	37.9	38.4	38.4	40.1	36.4	37.3
	**27.2**	**28.5**	**26.7**	**28.6**	**27.8**	**28.2**	**27.4**	**28.2**	**28.2**	**28.3**	**27.6**	**27.0**
**Seui (E2/IT)**	38.3	40.4	40.8	37.4	36.7	37.1	36.1	36.4	36.4	40.3	38.6	39.3
	**27.2**	**28.5**	**27.5**	**29.4**	**28.2**	**29.4**	**27.8**	**29.0**	**29.0**	**29.9**	**28.0**	**27.5**

SC: Scotland; USA: United States of America; SA: South-Africa; IC: Iceland; IT: Italy; LB: Lebanon; J: Jordan; GR: Greece; CH: China; SP: Spain; TR: Turkey; NW: Norway.

## Data Availability

The data presented in this study are available on request from the corresponding author. The data are not publicly available due to privacy restrictions.
